# Long Non-coding RNA X-Inactive Specific Transcript Mediates Cell Proliferation and Intrusion by Modulating the miR-497/Bcl-w Axis in Extranodal Natural Killer/T-cell Lymphoma

**DOI:** 10.3389/fcell.2020.599070

**Published:** 2020-12-08

**Authors:** Qinhua Liu, Ruonan Ran, Zhengsheng Wu, Xiaodan Li, Qingshu Zeng, Ruixiang Xia, Yalei Wang

**Affiliations:** ^1^Department of Hematology, The First Affiliated Hospital of Anhui Medical University, Hefei, China; ^2^Department of Gastroenterology, The First Affiliated Hospital of Anhui Medical University, Hefei, China; ^3^Department of Pathology, The First Affiliated Hospital of Anhui Medical University, Hefei, China

**Keywords:** ENKL, lncRNA, miRNA, proliferation, migration

## Abstract

The present study was directed toward laying new findings for Extranodal natural killer/T-cell lymphoma (ENKL)-oriented therapy with a focus on long non-coding RNA (lncRNA)–microRNAs (miRNAs)–mRNA interaction. The expression and function of XIST (X-inactive specific transcript) were analyzed both *in vivo* and *in vitro*. The online database of lncRNA-miRNA interaction was used to screen the target of XIST, and miR-497 was selected. Next, the predicted binding between XIST and miR-497, and the dynamic effect of XIST and miR-497 on downstream Bcl-w was evaluated. We found that XIST dramatically increased in the blood of ENKL patients and cell lines. XIST knockdown suppressed the cell proliferation and migration *in vivo* and *in vitro*. Herein, we confirmed the negative interaction between XIST and miR-497. Moreover, XIST knockdown reduced the protein levels of Bcl-w, a downstream target of miR-497. XIST sponges miR-497 to promote Bcl-w expression, and finally modulating ENKL cell proliferation and migration. To be interested, inhibition of Bcl-w by ABT737 can overcome the high expression of XIST, and suppressed the ENKL proliferation and migration by inducing apoptosis. This study provided a novel experimental basis for ENKL-oriented therapy with a focus on the lncRNA–miRNA–mRNA interaction.

## Introduction

Non-Hodgkin’s lymphoma (NHL) stems from B, T, and natural killer (NK) lymphocytes and comprises both indolent and aggressive types (Swerdlow et al., 2016). Extranodal NK/T-cell lymphoma (ENKL), nasal type, is an aggressive NHL representing a rare specific extranodal subtype of peripheral T-cell lymphoma (PTCL) (Tse and Kwong, 2017). ENKL occurs mainly (80%) in the nose, and upper aerodigestive tract, with only a small proportion (20%) observed in non-nasal areas (Au et al., 2009). ENKL is an aggressive lymphoma with poor survival in an untreated patient measured in weeks to months; the median overall survival is 3 years for localized disease and 8 months for metastatic disease (Lee et al., 2006). Currently, there is no single standard of care for the treatment of ENKL. Radiation and/or chemotherapy are generally used in all ENKL patients, but with very limited effects. Therefore, new molecular targeted therapies are required for the treatment of ENKL malignancies.

A recent study has indicated that most long non-coding RNAs (lncRNAs) possibly acts as molecular miRNAs sponges and release RNA transcripts that are targeted by active miRNAs (Wang and Chang, 2011). LncRNAs, comprising over 200 nucleotides, regulates gene expression through sponging miRNA and stabilizing mRNA (Batista and Chang, 2013). LncRNA expression is reduced in several cancers, confirming its oncogenic and anti-tumor effects, which, in turn, suggest that abnormal lncRNA expression is implicated in cancer progression to a considerable extent (Sanchez Calle et al., 2018). Nowadays, the lncRNA X-inactive-specific transcript (XIST) has garnered considerable attention, given that it is dramatically augmented in many types of cancer and might represent a promising oncogene (Sorensen et al., 2013). Moreover, XIST is transcribed from an X inactivation center that is directly connected with polycomb inhibitory complex 2, which controls histone H3 trimethylation at Lys27, inactivating the X chromosome (Ma et al., 2017). Regarding hematologic malignant tumors, lncRNAs have been well investigated in B-cell lymphoma/leukemia (Chen et al., 2017) but rarely researched in ENKL.

Here we investigated XIST expression level in ENKL patients and found that XIST was upregulated in these patients compared with healthy participants. We searched for downstream target miRNAs of XIST and found that miR-497/Bcl-w is a target of XIST. And miR-497 interacts with XIST, mediating ENKL cell proliferation and migration. Collectively, the present study laid a new experimental foundation for ENKL-oriented therapy with a focus on the lncRNA–miRNA–mRNA interaction.

## Materials and Methods

### Patients and Samples

A total of 54 cases of ENKL in the upper aerodigestive tract [34 males and 20 females; average age, 42 (range, 28–86) years] and 24 healthy donors [12 males and 12 females; average age, 41 (range, 34–61) years] were examined from the Hematology Department, the First Affiliated Hospital of Anhui Medical University. The detail information was list in Supplementary Table 1. The study was approved by the First Affiliated Hospital of Anhui Medical University and was conducted following relevant regulations and the Declaration of Helsinki. The study was approved to be conducted with obtaining informed consent from patients and donors. A small proportion of samples were collected to ensure the integrality of the remaining tissues. The relevant data were derived from the medical record library through a double-blind program and were analyzed anonymously so that the interest of patients would not be damaged.

### Cell Culture

The ENKL cell lines SNK-6 and SNT-8, previously reported by Nagata et al. (2001) were cultivated in RPMI 1640 containing 10% heat-inactivated human serum and 0.7 U/μL recombinant human IL-2 (rh IL-2). NK-92 and KHYG-1 cells were cultivated in α-MEM (containing horse serum and FBS, 12.5% of each, and 0.2 U/μL rh IL-2) and RPMI 1640 (containing 10% FBS and 0.1 U/μL rh IL-2). All cells were cultivated at 37°C under a 5% CO_2_ atmosphere.

### Lentiviral Infection, siRNA, and miRNA Transfection

Human XIST cDNA was obtained by amplification of SNK-6 cells. XIST cDNA and scrambled short hairpin RNA (shRNA) targeting XIST (GCCAACTGTCTGCTTAAGAAA) were linked to the LV-3 vector. HEK293T cells were covered with viruses as standardized schemes, incubated for 3 days, and used for infecting SNK-6, SNT-8, and NK-92 cells. The cells were treated with 2 μg/mL of puromycin for a fortnight to obtain steady transfected cells.

Lipofectamine 2000 was applied to the transfection of miR-497 mimics, antagomir, Bcl-w siRNA, and negative control (NC) into cells. The miRNA mimics, antagomir, and NC were prepared by GenePharma as previously reported (Zhang et al., 2018). The sequences for the siRNA of Bcl-w were GCAGACUUUGUAGGUUAUATT (siRNA 1) and GGC GGAGUUCACAGCUCUAUA (siRNA 2).

### Cell Proliferation Assay

Extranodal natural killer/T-cell lymphoma cell proliferation was determined using the cell counting Kit-8 (CCK8) and 5-Bromo-2′-deoxyUridine (BrdU) assays. An improved CCK8 assay was applied to assess cell viability. Cells were inoculated in 96-well plates (5 × 10^3^ cells/well) and transfected for 2 days before adding 0.1 mL CCK8; 4 h later, A_450_ was determined through enzyme labeling.

The BrdU assay was applied to determine cell proliferation using the Cell Proliferation Detection kit as per manufacturer’s instructions.

### Transwell Migration Assay

A cell suspension (0.2 mL) in FBS free medium was transferred to the upper transwell chambers (8 μm). RPMI-1640 (0.6 mL) with 10% FBS was added to the lower chambers. After 24 h of culture, viable ENKL cell numbers were counted on a hemocytometer by the Erythrosin B (Sigma Chemical Co., St. Louis, MO, United States) dye exclusion assay. The relative migration was calculated by comparing the migrated cell number with the control group.

### Quantitative Reverse Transcription Polymerase Chain Reaction (qRT-PCR)

Total RNA was isolated using RNAiso Plus. RNA was then reversely transcribed using Prime Script^TM^ RT Master Mix (Thermo Fisher, Waltham, MA, United States). qRT-PCR was performed using a PCR system; the primers used were as follows: for XIST (sense, 5′-AGCTCCTCGGACAGCTGTAA-3′; antisense, 5′-CTCCAGATAGCTGGCAACC-3′); for miR- 497-5p: (sense, 5′-CCTTCAGCAGCACACTGTGG-3′; antisense, 5′-CAGTGCAGGGTCCGAGGTAT-3′); for Bcl-w: (sense, CACCCAGGT CTCCGATGAAC; antisense, TTGTTG ACACTCTCA GCACAC); for GAPDH: (sense, 5′-CATGTT CCAATATGATTCCAC-3′; antisense, 5′-CCTGGAAGATGG TGATG-3′). The 2^–ΔΔCt^ method was used to investigate relative gene expression.

### Western Blot

Total protein was separated, lysed with 10% SDS-PAGE, and ultimately transferred to a PVDF membrane that was successively cultivated with primary and secondary antibodies. The main antibodies included Bcl-w (ab190952), Ki-67 (ab15580), glyceraldehyde 3-phosphate dehydrogenase (ab9485) (Abcam, Cambridge, Britain), E-cadherin (#3195), MMP7 (#71031), MMP9 (#13667), and vimentin (#5741) (Cell signaling, Danver, CO, United States). All the antibodies were diluted at 1:1000 in PBS with 5 % BSA. The second antibody for mouse (SC-2005) and rabbit (SC-2030) were purchased from Santa Cruz (Dallas, TX, United States), and diluted at 1: 4000 before used.

### Dual-Luciferase Reporter Assay (DLRA)

After amplification through PCR, the XIST fragment was cloned into the Renilla psiCHECK2 vector downstream as previously described (Liu et al., 2018), which was termed wt-XIST. The primers used were 5′-CCGCTCGAGcatagttctgttagttacac-3′, and 5′- AATGCGGCCGCatgcaattatgcatatcaac-3′, including XhoI and NotI. The XIST seed region underwent mutation to eliminate all miR-497 nucleotides 2–7 complementarity, thereby obtaining the XIST mutant reporter, which was termed mut-XIST. The primers for site mutation were 5′-tgtcctc tttgccatACGACGAgagttctgactaccc-3′, and 5′-gggtagtcagaactc TCGTCGTatggcaaagaggaca -3′. The Bcl-w wild type and mutated luciferase reporters were constructed as previously described (Yin et al., 2010). HEK293 cells were cultured in 24-well plates overnight, followed by co-transfection with the indicated vectors and miR-497 mimics. Two days later, LRA was performed using the DLRA System. Renilla luciferase activity was standardized with Firefly luciferase as the reference.

### RNA Immunoprecipitation (RIP)

RNA Immunoprecipitation was performed using the Magna RIP RNA-Binding Protein Immunoprecipitation Kit, and RNA for *in vitro* experiments was transcribed using the RNA Synthesis Kit as per manufacturer’s instructions. miR-497, IgG, and XIST levels in immunoprecipitates were measured via qRT-PCR.

### Animals

Balb/c athymic nude male mice (42 days old) were provided by Guangdong Medical Animal Center. All relevant operations were performed as per the NIH regulations regarding experimental animals’ use and care and were approved by Institutional Animal Care and Use Committee. The 2 × 10^6^ SNK-6 with or without XIST shRNA stable expression was washed, incubated in PBS, and subsequently injected into the lateral caudal vein or flank (0.1 mL). When the weight of the mice decreased to <20% of the original weight, they were executed. For flank xenografts, the tumor was harvested for Ki-67 staining. Six mice were used in each group of experiments. For caudal vein injection, the lungs of the executed mice were separated, followed by fixation with 10% formaldehyde solution. A dissecting microscope was used to observe the surface metastasis number in a single lung.

### Data Analysis

Data were analyzed using SPSS17.0 and expressed as average ± SD obtained from not less than three parallel experiments. Student’s *t*-test was used to analyze the statistical differences in means where applicable. Analysis of variance was used for estimating intergroup differences. ^∗^*P* < 0.05; ^∗∗^*P* < 0.01.

## Results

### XIST Expression in ENKL Patients

**FIGURE 1:**
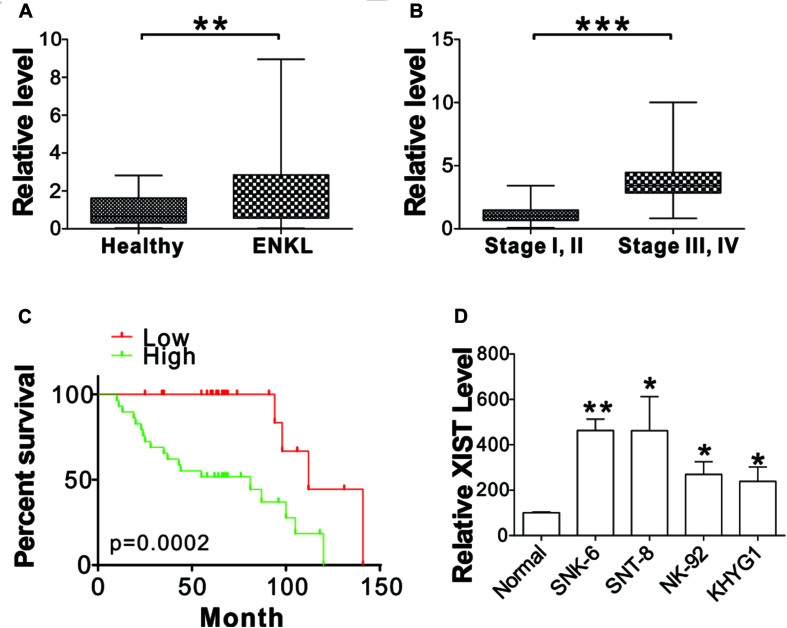
XIST is upregulated in ENKL cells. **(A)** The mRNA level of XIST in the blood of ENKL patients (ENKL, *n* = 54) and healthy donors (Healthy, *n* = 24). **(B)** The mRNA level of XIST in the blood of ENKL patients at different stages (Stage I, II, *n* = 41; Stage III, IV, *n* = 13). **(C)** The survival of ENKL patients with high and low XIST expression. **(D)** The mRNA level of XIST in normal PBM and ENKL cells, *n* = 3 for each sample. *p < 0.05; **p < 0.01; ***p < 0.001.

To study the role of XIST in ENKL, we first analyzed XIST mRNA levels in the blood sample collected from 54 ENKL patients and 24 healthy donors. The findings demonstrated that XIST expression was notably upregulated in ENKL patients (Figure 1A). ENKL patients were classified into two groups based on their clinical stages (*n* = 41 for stage I, II; *n* = 13 for stages III, IV). XIST expression levels were significantly higher in stage III and IV ENKL patients (Figure 1B). The 54 patients were further classified into high (higher than average, *n* = 29) and low (lower than average, *n* = 25) XIST expression groups. High XIST expression contributed to unfavorable clinical outcomes in these 54 ENKL patients (Figure 1C). To thoroughly explore the exact role of XIST that is involved in ENKL and the relevant molecular mechanism, PCR was used to determine XIST expression in four ENKL cell lines and one normal human PBMC line. XIST expression was dramatically augmented in ENKL cells, which was consistent with its high expression in the tissue samples (Figure 1D). Therefore, our data suggest that XIST is upregulated in ENKL patients and is correlated to poor patient survival.

### XIST Promotes ENKL Cell Proliferation and Migration

**FIGURE 2:**
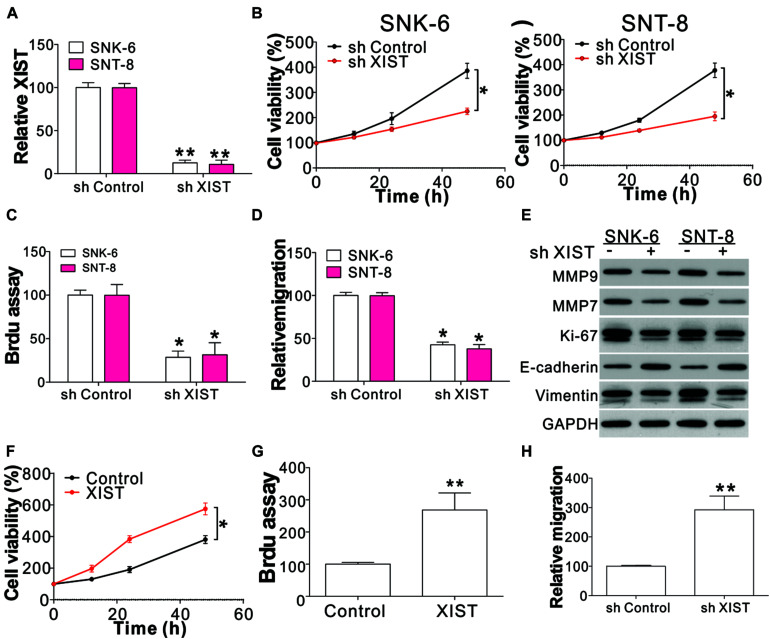
XIST mediates ENKL cell proliferation and migration. **(A)** The mRNA level of XIST in ENKL cells with a stably transfected control or XIST shRNA. **(B)** The viability of ENKL cells with a stably transfected control or XIST shRNA at different time points. **(C)** The BrdU assay of ENKL cells with a stably transfected control or XIST shRNA. **(D)** The transwell assay of ENKL cells with a stably transfected control or XIST shRNA. **(E)** The expression of indicated proteins in ENKL cells with stably transfected control or XIST shRNA. **(F)** The cell viability of NK-92 cells with a stably transfected control or XIST expression vector at different time points. **(G)** BrdU assay analysis of NK-92 cells with a stably transfected control or XIST expression vector. **(H)** Transwell assay analysis of NK-92 cells with a stably transfected control or XIST expression vector. Each experiment was repeated for 3 times. *p < 0.05; **p < 0.01.

To study the role of XIST on ENKL progression, XIST shRNA was transfected into SNK-6 and SNT-8 cells. The XIST expression was mirrored by real-time PCR (Figure 2A). Subsequently, the growth of the transfected cells was detected using BrdU and CCK8 assays. XIST knockdown notably inhibited cell proliferation (Figures 2B,C). The transwell assay revealed that the depletion of XIST suppressed cell migration (Figure 2D). We further analyzed the expression of cell proliferation, invasion, and migration markers including Ki-67, MMP7, MMP9, E-cadherin, and vimentin. Our data showed that the absence of XIST suppressed Ki-67, MMP7, MMP9, and vimentin expression but enhanced E-cadherin expression (Figure 2E and Supplementary Figure 1A). To further confirm the function of XIST involved in ENKL cell proliferation and migration, we infected XIST-expressing virus into NK-92 cells. We found that enhanced XIST expression in NK-92 cells had the opposite effect on cell proliferation and invasion (Figures 2F–H and Supplementary Figure 1B). XIST overexpression also suppressed E-cadherin expression increased Ki-67, MMP7, MMP9, and vimentin expressions (Supplementary Figure 1C). Therefore, these findings revealed that XIST is associated with ENKL proliferation and migration *in vitro*.

### Knocking Down XIST Inhibits ENKL Tumor Growth and Metastasis *in vivo*

**FIGURE 3:**
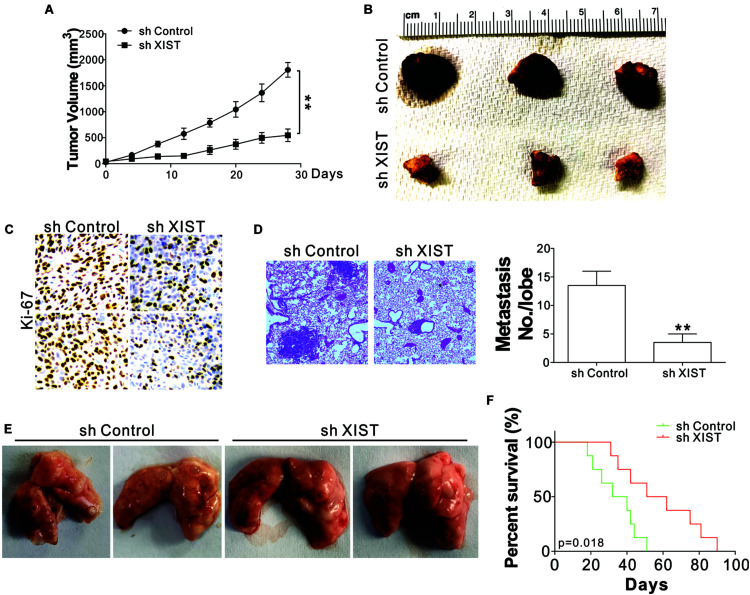
Depletion of XIST suppressed SNK-6 xenograft growth and metastasis. **(A)** The tumor growth in nude mice xenografted with SNK-6 stably transfected with a control or XIST shRNA (*n* = 6). **(B)** Representative tumors. **(C)** Representative Ki-67 staining in different tumor groups. **(D)** The H&E staining and metastatic number in the lung of nude mice injected with SNK-6 stably transfected with a control or XIST shRNA via tail vein at 5 weeks. **(E)** Representative pictures of lung tumors in different groups of mice (*n* = 6). **(F)** The survival of nude mice injected with SNK-6 stably transfected with a control or XIST shRNA via tail vein (*n* = 8). **p < 0.01.

To confirm that XIST plays a role in the growth of ENKL *in vivo*, the growth of SNK-6 xenografts in nude mice was examined. SNK-6 cells with or without stable XIST knockdown were inoculated subcutaneously into nude mice. The tumors with XIST knockdown had a slower growth rate and a smaller size than those with control shRNA transfection (Figures 3A,B). Furthermore, Ki-67 staining revealed that the depletion of XIST suppressed tumor proliferation (Figure 3C). A tumor metastasis model by tail-vein injection of tumor cells was used to investigate whether XIST promotes metastasis. SNK-6 cells with or without stable XIST knockdown were injected into nude mice via tail vein injection. Five weeks after the injection, the mice were sacrificed to observe tumor formation in the lungs. Metastatic tumors were present and verified through H&E staining of sectioned lung tissue excised from the mice. We found that the tumor size and number were smaller in the mice injected with stable XIST knockdown SNK-6 cells (Figures 3D,E). Without XIST in tumor cells, the mice exhibited better survival (Figure 3F). Collectively, these results indicate that XIST expression mediates ENKL growth and metastasis *in vivo*.

### XIST Promotes ENKL Proliferation and Migration by Targeting miR-497

**FIGURE 4:**
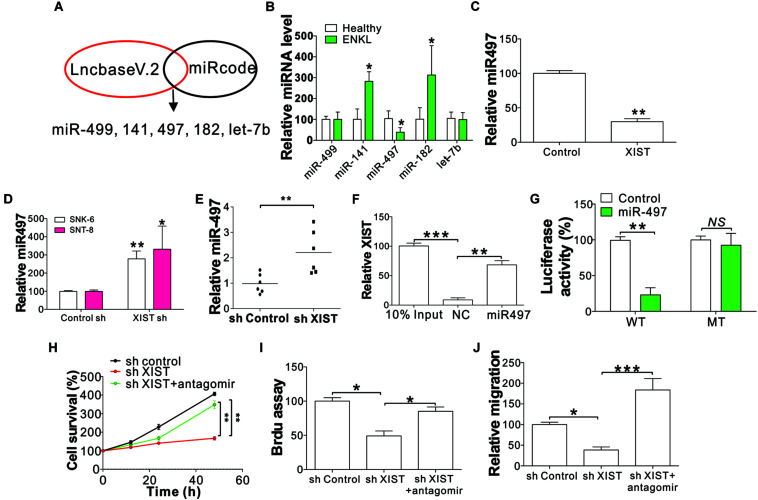
XIST-mediated ENKL cell proliferation and migration following targeting of miR-497. **(A)** The process of search for XIST-targeting miRNAs. **(B)** The expression of indicated miRNAs in ENKL patients and healthy donors. **(C)** The expression of miR-497 in NK-92 cells stably expressing XIST. **(D)** The expression of miR-497 in SNK-6 and SNT-8 cells stably transfected with a control or XIST shRNA. **(E)** The expression of miR-497 in tumors of mice xenografted with SNK-6 stably transfected with a control or XIST shRNA (*n* = 6). **(F)** The interaction of XIST and miR-497 analyzed via biotin-label miR-497 pull down. **(G)** The luciferase reporter activity of wild-type (WT) or mutant XIST fragment-constructed reporters with miR-497 co-transfection. The effect of miR497 on luciferase activity was normalized to control RNA transfection in each group. **(H)** The viability of SNK-6 cells with a stably transfected control or XIST shRNA in combination with the transfection of the miR-497 antagomir. **(I)** BrdU assay analysis of SNK-6 cells with a stably transfected control or XIST shRNA in combination with the transfection of the miR-497 antagomir. **(J)** Transwell assay analysis of SNK-6 cells with a stably transfected control or XIST shRNA in combination with the transfection of the miR-497 antagomir. Each experiment was repeated for 3 times. NS, *p* > 0.05; *p < 0.05; **p < 0.01; ***p < 0.001.

LncRNAs generally function by interacting with downstream target miRNAs (Paraskevopoulou and Hatzigeorgiou, 2016). To explore whether XIST regulates the proliferation and migration of ENKL cells by interacting with miRNAs, we scanned the online databases Mircod^1^ and lncBase v.2^2^ for XIST interacting miRNAs. We selected the top five miRNAs in the overlap of these two databases, including miR-499, miR-141, miR-497, miR-182, and let-7b (Figure 4A). Next, we analyzed the expression of these five miRNAs in SNK-6 cells with XIST knockdown or overexpression. We found only miR-497 expression in ENKL patients was downregulated compared with that in healthy donors (Figure 4B), showing that miR-497 is a likely target of XIST. We further analyzed miR-497 mRNA levels in ENKL cells with XIST overexpression or knockdown. Our results revealed that XIST overexpression suppressed miR-497 in NK-92 cells (Figure 4C), but XIST knockdown enhanced miR-497 expression in ENKL cells (Figure 4D). The miR-497 expression was also higher in the xenografted tumors with XIST knockdown (Figure 4E). To further verify miR-497 as a target of XIST, a specific biotin-marked miR-497 probe successfully captured XIST (Figure 4F). Dual-luciferase reporter assay (DLRA) was also used to investigate the direct relevance of miR-497 and XIST according to their complementary sequences. Wild-type (WT) or mutant XIST fragments were established and introduced into the luciferase reporter gene downstream. Next, the miR-497 mimic with the reporter gene was co-transfected into SNK-6 cells. A noticeable decrease in WT luciferase reporter activity was detected in relation to control RNA co-transfection (Figure 4G). However, miR-497 co-transfection did not affect mutant luciferase reporter activity (Figure 4G). Furthermore, the expression of XIST in ENKL patients was negatively correlated with the expression of miR-497 (Supplementary Figure 2A). Obviously, a direct relationship between XIST and miR-497 was verified.

To further investigate whether miR-497 is necessary for ENKL proliferation and migration driven by XIST, we transfected the stable XIST knockdown SNK-6 cells with miR-497 antagomir. Consistent with our hypothesis, miR-497 inhibition counteracted the suppressive effect of XIST depletion on the proliferation and migration of ENKL cells (Figures 4H–J). Furthermore, transfection of the miR-497 mimic in SNK-6 and SNT-8 cells phenocopied the effect of XIST depletion, which also suppressed the proliferation and migration of ENKL cells (Supplementary Figures 2B–D). Collectively, these findings indicate that miR-497 is the downstream target of XIST in the proliferation and migration of ENKL cells.

### Bcl-w Is a Direct Target of miR-497 in ENKL Cells

**FIGURE 5:**
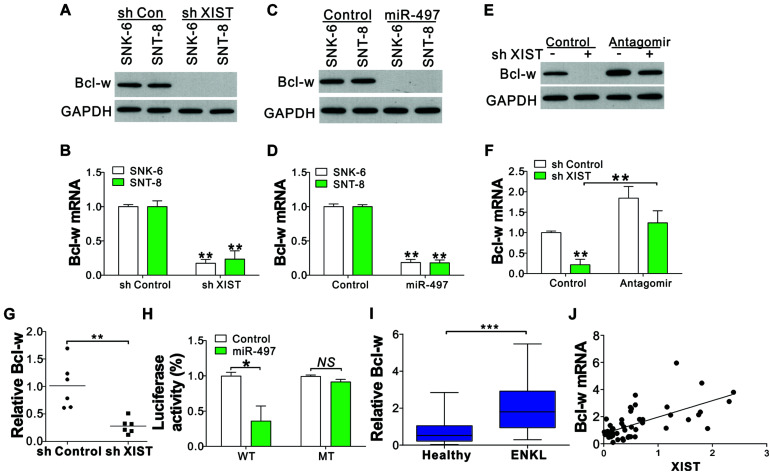
XIST/miR-497 modulates Bcl-w expression in ENKL cells. **(A,B)** The protein **(A)** and mRNA **(B)** levels of Bcl-w in ENKL cells stably treated with a control or XIST shRNA. **(C,D)** The protein **(C)** and mRNA **(D)** levels of Bcl-w in ENKL cells treated with an miR-497 mimic. **(E,F)** The protein **(E)** and mRNA **(F)** levels of Bcl-w in SNK-6 cells stably transfected with a control or XIST shRNA with or without miR-497 antagomir transfection. **(G)** The mRNA expression of Bcl-w in tumors of mice xenografted with SNK-6 stably transfected with a control or XIST shRNA (*n* = 6). **(H)** The luciferase reporter activity of a wild-type (WT) or mutant Bcl-w reporter in SNK-6 cells stably transfected with a control or miR-497. The effect of miR497 on luciferase activity was normalized to control RNA transfection in each group. **(I)** The mRNA level of Bcl-w in ENKL patients (*n* = 54) and healthy donors (*n* = 24). **(J)** The correlation of Bcl-w mRNA and XIST expression in 54 ENKL patients. Each experiment was repeated for 3 times. NS, *p* > 0.05; *p < 0.05; **p < 0.01; ***p < 0.001.

Previously, miR-497 has been identified as a tumor inhibitor based on its effects on Bcl-w, (Shen et al., 2012) an anti-apoptosis Bcl-2 protein that plays an essential part in cell survival. Here we investigated whether Bcl-w could be modulated by the XIST/miR-497 axis. We found that Bcl-w was downregulated in XIST shRNA-transfected ENKL cells (Figures 5A,B). Transfection of miR-497 mimic into ENKL cells also suppressed Bcl-w protein and mRNA levels (Figures 5C,D). Conversely, transfection of miR-497 antagomir abolished the suppressive effect of XIST depletion on Bcl-w expression (Figures 5E,F). The mRNA level of Bcl-w was also lower in the xenografted tumors with XIST knockdown (Figure 5G). Furthermore, we co-transfected the Bcl-w luciferase reporter vector with miR-497 or control miRNA into SNK-6 cells and determined the luciferase activity. The results showed that miR-497 considerably suppressed luciferase activity relative to NC miRNA (Figure 5H). However, miR-497 did not suppress the luciferase activity of the reporter vector containing Bcl-w 3′-UTR with a three-point mutation in the miR-497-connecting site (Figure 5H). Furthermore, the mRNA level of Bcl-w in primary ENKL tumors was also higher than that in healthy tissues (Figure 5I) and was positively correlated with the XIST expression level in the 54 ENKL patients (Figure 5J). These findings revealed that miR-497 specifically inhibits Bcl-w protein synthesis in ENKL cells.

### Deletion of Bcl-w Suppresses XIST-Mediated ENKL Proliferation and Migration

**FIGURE 6:**
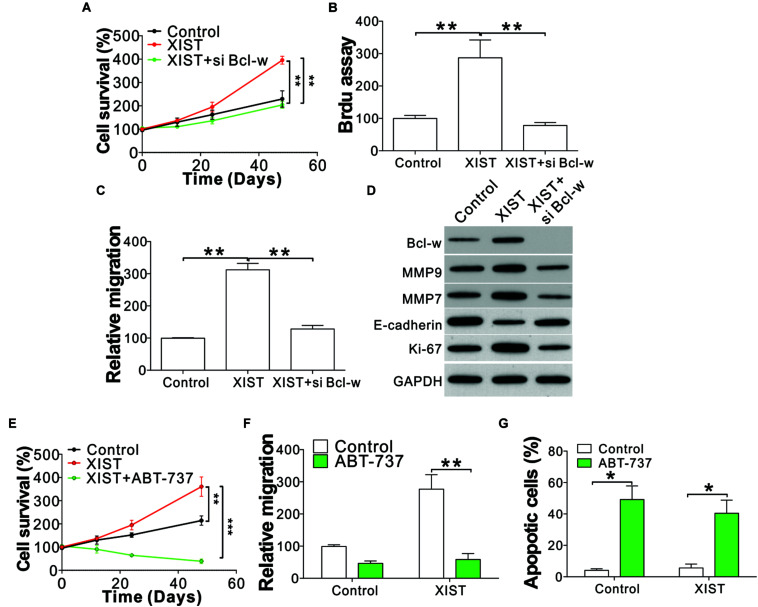
Deletion or inhibition of Bcl-w suppressed the proliferation and migration of ENKL cells with high XIST expression. **(A)** The cell viability of NK-92 cells with a stably transfected control or XIST expression vector with co-transfection of Bcl-w siRNA. **(B)** BrdU assay analysis of NK-92 cells with a stably transfected control or XIST expression vector with co-transfection of Bcl-w siRNA. **(C)** Transwell assay analysis of NK-92 cells with a stably transfected control or XIST expression vector with co-transfection of Bcl-w siRNA. **(D)** The indicated protein expression in NK-92 cells with a stably transfected control or XIST expression vector with co-transfection of Bcl-w siRNA. **(E)** The cell viability of NK-92 cells with stably transfected control or XIST expression vector with or without ABT-737 (4 μM) treatment. **(F)** The migration of NK-92 cells as treated in panel **(E)**. **(G)** Hoechst-33258 staining of the nucleus for apoptosis in NK-92 cells as treated in panel **(E)**. Each experiment was repeated for 3 times. *p < 0.05; **p < 0.01; ***p < 0.001.

We next examined the function of Bcl-w in XIST-mediated ENKL cell proliferation and migration. Silencing of Bcl-w by siRNA suppressed NK-92 cell proliferation and migration with or without XIST overexpression (Figures 6A–D and Supplementary Figures 3A–C). We then investigated whether the Bcl-w inhibitor ABT-737 could be used to overcome the ENKL cell proliferation and migration induced by XIST overexpression. Similar to Bcl-w knockdown, ABT-737 treatment suppressed NK-92 cell proliferation and migration, even with XIST overexpression (Figures 6E,F). ABT-737 not only suppressed NK-92 cell proliferation but also caused cell loss (Figure 6E). ABT-737 has been reported to induce apoptosis in multiple cancer cells, and we also found apoptotic signals in ABT-737-treated NK-92 cells using nucleus fragmentation stained with Hoechst-33258 (Figure 6G). Therefore, our results indicated that miR-497 acts on Bcl-w to modulate ENKL proliferation induced by XIST.

## Discussion

Currently, abnormal lncRNA expression has been extensively detected and confirmed to be related to tumor occurrence. LncRNA XIST is reportedly able to accelerate metastasis through targeting miRNAs (Chen et al., 2017). Likewise, it has been shown to facilitate colon cancer development through the Wnt/β-catenin signaling pathway (Sun et al., 2018). Although research on XIST as an oncogene is rapidly increasing, no study has examined the role of XIST in ENKL patients. In the present study, we found increased XIST expression in the ENKL tissues of 54 patients; more importantly, high XIST expression was found to be associated with unfavorable prognosis, suggesting an oncogenic component of XIST involved in ENKL pathology.

LncRNAs have garnered increased attention because of their essential roles in normal physiological and pathogenic processes (Li et al., 2017). XIST has been reported to be involved in the biological processes of cancer cells, and it is resistant to chemo- and radiotherapies. XIST can influence gastric cancer cell proliferation, migration, intrusion, and tumor growth by sponging miR-101 (Chen et al., 2016). XIST can also regulate the biological processes of bladder cancer cells by acting on miR-124 (Xiong et al., 2017). The present work indicated that XIST knockdown notably inhibited ENKL cell proliferation and migration, which agrees with the conclusions from other studies.

It has been extensively acknowledged that lncRNA–miRNA–mRNA interactions assume an essential part in tumor occurrence, (Zhou et al., 2019) and previous studies have indicated that XIST plays an important role in cancers mainly by interacting with diverse miRNAs (Esquela-Kerscher and Slack, 2006). To thoroughly explore the mechanism of XIST in ENKL, we scanned the miRNA–lncRNA interaction database and selected the top five predicted miRNAs. We further analyzed the expression of these miRNAs in ENKL patients and found that miR-497 was downregulated. Furthermore, we verified that miR-497 is the downstream target of XIST, which mediates ENKL cell proliferation and migration. The dysregulation of miRNAs can trigger diverse cancers, including malignant lymphocytic tumors (Peng and Croce, 2016). Most known and characterized miRNAs are thought to act as tumor inhibitors or oncogenes, and their respective function is dependent on the cell type or has a tissue-specific context (Fabbri et al., 2008). MiR-497 has been reported to act as a tumor suppressor in multiple cancers, including lymphoma (Troppan et al., 2015). Furthermore, miR-497 has been reported to be the target of XIST in gastric cancer and hepatocellular carcinoma (Zhu et al., 2019). Consistent with previous studies, we also found that miR-497 suppressed the XIST-induced ENKL tumor growth and metastasis, which is an important target in patients with high XIST expression.

Regarding miR-497 downstream transcripts, we found that Bcl-w is the downstream transcript target, which could be regulated through XIST/miR-497 axis. Impaired apoptosis signaling is a precondition for tumor occurrence (Zhang et al., 2019). And the anti-apoptosis BCL-2 family proteins are major cell survival modulators that are frequently overexpressed in malignant tumors, resulting in augmented cancer cell survival (Gibson et al., 1996; Adams et al., 2017). Different from BCL-XL and BCL-2, the closest anti-apoptosis relative BCL-W is warranted for sperm development but not for other types (Hata et al., 2015). In recent years, several studies have revealed that Bcl-w is essential for B-cell survival and lymphomagenesis (Gibson et al., 1996). Bcl-w deprivation substantially inhibited MYC-modulated B-cell lymphoma occurrence owing to enhanced MYC-triggered B-cell apoptosis. Beyond cell death, pro-survival proteins from the BCL-2 family have been shown to contribute to the migratory ability of cancer cells, (Um, 2016; Gross and Katz, 2017) and the role of Bcl-w to this process has been delineated. It is reasonable that the anti-apoptotic function of Bcl-w contributes to survival of drug-resistant cells. As to the non-apoptotic role of Bcl-w, it was reported to enhance the migratory and invasive potentials of gastric cancer cells by facilitating the production of several types of extracellular matrix (ECM)-degrading proteinases (Bhatnagar et al., 2010). Secreted matrix metallopeptidase-2 (MMP-2) and urokinase-type plasminogen activator surface receptor (uPAR) have been demonstrated to activate focal adhesion kinase (FAK), which acts as an executioner of Bcl-w-dependent invasive phenotype of gastric cancer cells (Bae et al., 2009). In this study, we found that ENKL patients exhibit high expression of Bcl-w, which is modulated by XIST/miR-497 signals. Furthermore, our data suggested that dysregulation of Bcl-W in ENKL promotes the cancer cell proliferation and migration. These findings reveal a novel mechanism for the dysregulation of Bcl-w in lymphomas, and targeting Bcl-w might be a potential strategy to overcome the ENKL progression and metastasis.

In summary, XIST acts as a miR-497 ceRNA by sponging miR-497, fighting with Bcl-w for connection with miR-497, and ultimately regulates ENKL cell proliferation and metastasis. Taken together, these findings indicate that XIST markedly promotes ENKL progression and that targeting the XIST/miR-497 axis can be a potential strategy to overcome the high cell proliferation and migration induced by abnormal Bcl-w expression.

## Data Availability Statement

The original contributions presented in the study are included in the article/Supplementary Material, further inquiries can be directed to the corresponding authors.

## Ethics Statement

The studies involving human participants were reviewed and approved by the study was approved by the First Affiliated Hospital of Anhui Medical University and was conducted following relevant regulations and the Declaration of Helsinki. Written informed consent for participation was not required for this study in accordance with the national legislation and the institutional requirements.

## Author Contributions

QL and RX conceived the study and designed the experiments. RR and ZW contributed to the data collection, XL and QZ performed the data analysis and interpreted the results. QL wrote the manuscript. RX and YW contributed to the critical revision of article. All authors read and approved the final manuscript.

## Conflict of Interest

The authors declare that the research was conducted in the absence of any commercial or financial relationships that could be construed as a potential conflict of interest.

## Footnotes

1 http://www.mircode.org/

2 http://carolina.imis.athena-innovation.gr/diana_tools/web/index.php?r=lncbasev2/index
