# Supplementary material for: Long Non-coding RNA X-Inactive Specific Transcript Mediates Cell Proliferation and Intrusion by Modulating the miR-497/Bcl-w Axis in Extranodal Natural Killer/T-cell Lymphoma
Source: Front Cell Dev Biol. 2020 Dec 8;8:599070. doi: 10.3389/fcell.2020.599070 (PMC7753184; doi:10.3389/fcell.2020.599070)

Fig. S1. Enhanced XIST expression in NK-92 promotes cell proliferation and migration. (A) The statistic analysis of indicated proteins expression in SNTK-6 cells transfected with control or XIST shRNA. (B) The mRNA level of XIST in NK-92 cells stably transfected with a control or XIST vector. (C) The indicated protein expression in NK-92 cells stably transfected with a control or XIST vector. Each experiment was repeated for 3 times. *, $p<0.05$; **, $p<0.01$.

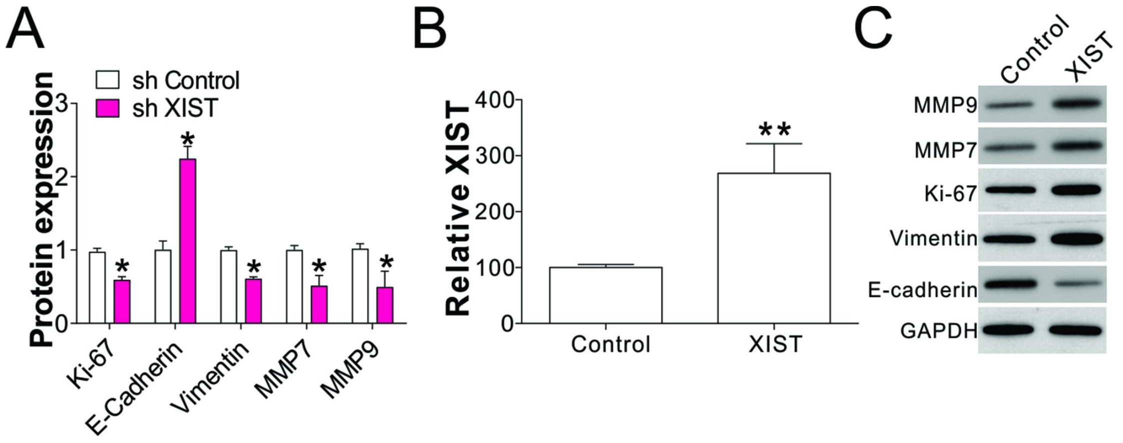

Fig. S2. The mimic of miR-497 suppressed ENKL cell proliferation and migration. (A) The correlation of miR-497 and XIST expression in 54 ENKL patients. SNK-6 and SNT-8 cells were transfected with either a control or miR-497 mimic. The cell proliferation and migration was analyzed via CCK8 assay (B), BrdU assay (C), and transwell assay (D). Each experiment was repeated for 3 times. *, p<0.05; **, p<0.01.

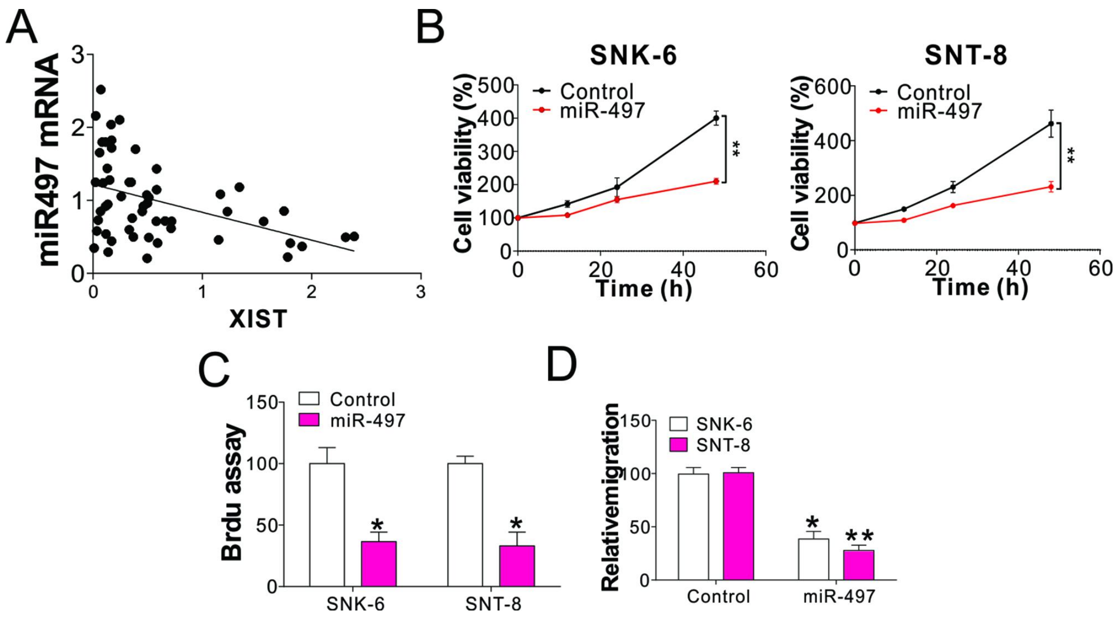

Fig. S3. Knockdown of Bcl-w suppressed the proliferation and migration of ENKL cells with high XIST expression. The NK-92 cells with a stably transfected control or XIST expression vector were transfected with control or alternative Bcl-w siRNA (Bcl-w siRNA 2). (A) The expression of Bcl-w. (B) BrdU assay analysis of proliferation. (C) Transwell assay analysis of cell migration. Each experiment was repeated for 3 times. **, p<0.01.

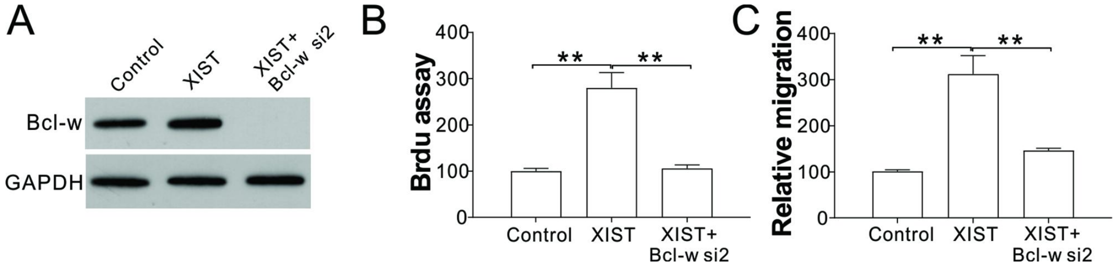

Supplement: Supplementary file 1 [file Data_Sheet_1.pdf]
