# Supplementary material for: Long Non-coding RNA X-Inactive Specific Transcript Mediates Cell Proliferation and Intrusion by Modulating the miR-497/Bcl-w Axis in Extranodal Natural Killer/T-cell Lymphoma
Source: Front Cell Dev Biol. 2020 Dec 8;8:599070. doi: 10.3389/fcell.2020.599070 (PMC7753184; doi:10.3389/fcell.2020.599070)
Supplement: Supplementary file 2 [file Table_1.docx]

**Table 1. The information of ENKL patients and healthy donors.**
